# First Identification of a Heterozygous *RNU4‐2* and *RNU4‐1* Deletion Associated With Fetal Urogenital and Anorectal Malformations

**DOI:** 10.1002/pd.70138

**Published:** 2026-03-20

**Authors:** Mario Abaji, Bénédicte Gerard, Laurent Nasca, Radia Fritih, Julie Blanc, Pierre Castel, Claude D'Ercole, Annie Levy‐Mozziconacci

**Affiliations:** ^1^ Centre Pluridisciplinaire de Diagnostic Prénatal (CPDPN) Hôpital Nord AP‐HM Marseille France; ^2^ Aix Marseille Univ INSERM MMG Marseille France; ^3^ Laboratoire Eurofins Biomnis Lyon France

**Keywords:** prenatal diagnosis, ReNU syndrome, RNU4‐1, RNU4‐2, VATER/VACTERL association

## Abstract

- What is already known about this topic?
  ◦ Pathogenic sequence variants in *RNU4‐2* cause ReNU syndrome characterized by severe neurodevelopmental disorder, microcephaly, and brain malformations.
  ◦ This syndrome has been recently described in hundreds of individuals. Urogenital malformations have not been described as part of the ReNU syndrome spectrum.
- What does this study add?
  ◦ We report the first identification of a complete deletion encompassing *RNU4‐2*/*RNU4‐1* genes, presenting with bilateral renal and bladder agenesis, anorectal atresia, and vertebral anomalies resembling VATER/VACTERL association.
  ◦ This case suggests U4 snRNA haploinsufficiency as a candidate mechanistic hypothesis for these anomalies, which are markedly distinct from the phenotype associated with pathogenic sequence variants in *RNU4‐2.*

## 1 Fetal Phenotype

A 16‐week pregnancy was referred for prenatal consultation following detection of severe fetal anomalies on routine ultrasound. Detailed ultrasound examination revealed complete anhydramnios with non‐visualization of both kidneys and bladder. Following medical termination at 18 weeks plus 6 days of gestational age (GA), fetal examination was conducted on the male fetus demonstrating complete bilateral renal agenesis with absent bladder, consistent with the Potter sequence. Gastrointestinal examination revealed anorectal atresia with rectal ampulla atresia and anal imperforation. A single umbilical artery was identified. Skeletal examination demonstrated vertebral anomalies, including mid‐thoracic synostosis, lower thoracic vertebral body fusion, and sacral abnormalities. This phenotype fulfilled the diagnostic criteria for VATER/VACTERL (vertebral, anal, cardiac, tracheo‐esophageal, renal, and limb abnormalities) association (Table 1A).

**TABLE 1A:** Clinical data: GA (Gestational age), TOP (Termination of pregnancy).

Case	Parental details			Gestation at diagnosis	Phenotypes (HPO terms)	Obstetric history	Family history	Outcome
Pregnancy	Maternal	Age	33	18 GA+3	‐Renal agenesis (HP:0,000,104) ‐Anhydramnios (HP:0,025,700) ‐Potter facies (HP:0,002,009) ‐Anal atresia (HP:0,002,023) ‐Anorectal anomaly (HP:0,012,732) ‐Vertebral segmentation defect (HP:0,003,422) ‐ Single umbilical artery (HP:0,001,195)	G1P0	Interatrial communication (Mother)	TOP
		Ethnicity	European					
	Paternal	Age	32					
		Ethnicity	European					

## 2 Diagnostic Method

Chromosomal microarray analysis (CMA) performed on DNA extracted from fetal biopsy (lung) was normal. Trio exome sequencing was then performed and analyzed at the Eurofins Biomnis laboratory using massive parallel sequencing with the Human Exome 2.0 Plus Comprehensive Exome kit. Sequencing was performed on the Illumina NovaSeq 6000. Variants were identified and annotated using an external pipeline (SeqOne, Hg19), while CNV (Copy Number Variation) analysis was conducted using both SeqOne and local pipelines.

## 3 Diagnostic Results and Interpretation

Trio exome sequencing identified a de novo heterozygous deletion of 48 kb in the 12q24.31 chromosomal region (chr12:g.120703070_120751189del, GRCh37). This deletion encompasses *RNU4‐2, RNU4‐1, SIRT4*, and the first exon of *PXN*. Of note, current exome capture does not include probes targeting the non‐coding *RNU4‐2* and *RNU4‐1* genes, but this deletion was detected because it encompassed part of the neighboring *SIRT4* and *PXN* genes. It was classified as a variant of uncertain significance (VUS) because of the absence of other reported cases with similar deletions and the divergent phenotype from established ReNU syndrome (RNU4‐2 related syndrome). Furthermore, haploinsufficiency has not been implicated in the pathogenesis of this gene (Table 1B).

**TABLE 1B:** Genetic findings: NA (Not Applicable).

Procedure (Gest age)	Direct/culture?	Performed test	Gene (name; REFSEQ)	Known disease (OMIM)	Variant	ACMG classify‐cation	Inheritance & zygosity	Interpret‐ation
Fetal biopsy at (18 GA+6) lung tissue	Direct	Trio exome sequencing	*RNU4‐2* *RNU4‐1* *SIRT4* *PXN*	‐ReNU syndrome (#620851) ‐NA ‐NA ‐NA	chr12:g.120703070_120751189del, GRCh37	VUS (Class 3)	De novo heterozygous	Possible implication in fetal phenotype

## 4 Discussion

We report a complete heterozygous deletion encompassing *RNU4‐2*/*RNU4‐1*, the two human U4 small nuclear RNA genes. U4 snRNA is an essential component of the major spliceosome and is required for splicing over 99% of introns [1]. This case raises important questions about haploinsufficiency in snRNA genes beyond the established ReNU syndrome phenotype.

Pathogenic sequence variants in *RNU4‐2* cause ReNU syndrome, characterized by severe neurodevelopmental disorder, microcephaly, and brain malformations [2, 3, 4]. While pathogenic sequence variants likely retain residual snRNA function, complete deletion results in a reduction in U4 snRNA dosage that may be below the threshold required for normal organogenesis. This may affect early developmental programs differently than pathogenic sequence variants. Urogenital and gastrointestinal systems, which undergo critical morphogenesis during the first trimester, may be particularly vulnerable to reduced snRNA levels during organogenesis when splicing demands are high. Indeed, in other spliceosomopathies, haploinsufficiency cannot sustain the massive transcriptional burden of rapid embryonic growth, resulting in targeted malformations [5]. The anomalies in this case resemble the VATER/VACTERL association, for which the genetic etiology remains unknown in most cases [6]. This suggests U4 snRNA haploinsufficiency as a candidate mechanistic hypothesis for these anomalies, though a contributing role for the neighboring *PXN* and *SIRT4* genes cannot be completely excluded.

The absence of replicated cases with similar deletions and the divergent phenotype from known ReNU syndrome precluded pathogenic classification. However, identifying additional cases is crucial not only to establish causality but also to understand the full phenotypic spectrum of *RNU4‐2/RNU4‐1* haploinsufficiency. We strongly encourage clinicians and laboratories to reexamine this genomic region in any severe congenital anomaly case, especially when genome sequencing is available, as this deletion may not be detected using standard care testing. Cases with deletions, even if presenting with different phenotypes, would be invaluable for refining genotype‐phenotype correlations and understanding dosage sensitivity of snRNA genes. Reporting such cases will enable variant reclassification.

## Funding

The authors have nothing to report.

## Ethics Statement

This study was approved by the Ethics and Scientific Committee (Comité d’Éthique et Scientifique, CSE) of AP‐HM under approval number N° CSE25‐394. The committee reviewed the project on 02/12/2025, and issued a favorable opinion after evaluating its scientific rationale, feasibility, patient information process, and ethical considerations.

## Consent

The couple consented for publication.

## Conflicts of Interest

The authors declare no conflicts of interest.

## Data Availability

The data that support the findings of this study are available from the corresponding author upon reasonable request.
